# Comparison of digital subtraction angiography combined arterial thrombectomy versus simple arterial thrombectomy in the treatment of acute lower limb ischemia

**DOI:** 10.1186/s12893-021-01297-x

**Published:** 2021-07-15

**Authors:** Hongwei Ge, Bin Song, Xin Wang, Yunfeng Zhu, Yiming Huang, Weibin Huang, Yongbin Zhu

**Affiliations:** Department of Vascular Surgery, The Third Affiliated Hospital of Soochow University, No.185 Juqian Street, Tianning District, Changzhou, 213003 Jiangsu China

**Keywords:** Arterial thrombectomy, Acute lower limb ischemia, Digital subtraction angiography, Mortality

## Abstract

**Background:**

This study aimed to compare the clinical efficacy of digital subtraction angiography (DSA) combined arterial thrombectomy versus simple arterial thrombectomy in the treatment of acute lower limb ischemia (ALI).

**Methods:**

This retrospective cohort study collected the clinical data from 124 patients (128 affected lower limbs) with ALI who underwent emergency surgery from March 2010 to November 2019. Patients were consecutively divided into Group A and Group B. Patients in Group A underwent simple arterial thrombectomy via the Fogarty catheterization. Patients in Group B underwent arterial thrombectomy, and the DSA was performed during the surgery. The differences in the success rate of primary surgery, the second intervention rate, and the amputation/mortality rate within 30-days after surgery were compared.

**Results:**

In Group A, 4 of 70 limbs (5.7%) were amputated, 54 of 70 limbs (77.1%) had improved blood flow, 14 of 70 limbs (20.0%) received a second intervention, and 3 of 68 patients (4.4%) died within 30 days. In Group B, 1 of 58 limbs (1.7%) was amputated, 56 of 58 limbs (96.6%) had improved blood flow, 3 of 58 limbs (5.2%) received a second intervention, and 2 of 56 patients (3.5%) died within 30-days. The success rate of primary surgery, the second intervention rate, and the amputation rate of Group B were significantly lower than Group A (*P* < 0.05).

**Conclusion:**

Arterial thrombectomy combined with DSA may effectively improve the clinical efficacy of patients with ALI.

## Background

Acute lower limb ischemia (ALI) is the sudden decrease in blood perfusion of lower limbs due to many causes, such as embolic and thrombotic vascular occlusion [1]. The annual amputation rate ranges is high as 20%-30% and the annual morbidity of ALI ranges from 1/1000 to 1.5/1000, which seriously threatens the survival of patients [2–4]. ALI patients are usually associated with “6 P” signs, including pain, pulselessness, pallor, decreased skin temperature, numbness, and dyskinesia and their good prognosis is depended on the rapid diagnosis and prompt and effective treatment [5]. Currently, the treatment methods of ALI mainly include arterial thrombectomy and catheter contact thrombolysis (CDT), percutaneous mechanical thrombolysis (PMT), and thrombolytic aspiration [6]. Among these methods, the arterial thrombectomy can remove embolization foci, restore blood supply of limbs, and prevent further injury by limb ischemia in a short time, while its disadvantage is the high incidence of ischemia/reperfusion (I/R) injury. The intraoperative fluoroscopy and fluoroscopic arteriotomy proposed by Parsons et al*. *[7] have improved the success rate of thrombectomy to a great extent. Along with the popularity of the digital subtraction angiography (DSA) hybrid operating room, more and more cases receive surgical treatment under DSA at present, which also obviously increases the success rate of ALI treatment [8].

In this study, we selected the patients with ALI who underwent DSA combined arterial thrombectomy or simple arterial thrombectomy from March 2010 to November 2019 in our hospital. We retrospectively compared and reported the differences in the clinical efficacy of the two methods in the treatment of ALI.

## Methods

### Clinical data

This study was approved by the Ethics Committee of the Third Affiliated Hospital of Soochow University, Patient informed consent was obtained for the study. We collected the clinical data from 124 patients with ALI who underwent DSA combined arterial thrombectomy or simple arterial thrombectomy from March 2010 to November 2019 in our hospital. There were 128 affected lower limbs of the 125 patients. Inclusion criteria: admission of acute non-traumatic disease < 14 days; manifestation of low limb: pain, pallor, anesthesia, paralysis, pulselessness. Artery occlusion was confirmed by color doppler ultrasound and computed tomography angiography (CTA). Critical limb ischemia (CLI) caused by graft and stent thrombosis and aortic dissection was not included. Exclusion criteria: Accompanied by severe cardiopulmonary dysfunction, coagulation dysfunction, liver and kidney failure which cannot suffer from surgery, advanced malignant tumor, etc. Table 1 summarizes the demographic data of patients in the different treatment groups.

**Table 1:** Patient demographics (124 patients)

	Group A (n = 68)	Group B (n = 56)	*t*/${x}^{2}$	*P*
Age ($\stackrel{-}{x}$±*s*, y)	67.35 ± 5.40	69.93 ± 7.93	1.45	0.17
Gender (M/F)	42/26	36/20	0.08	0.77
Chronic ischemic coronaropathy, n (%)	53 (77.9)	46 (82.1)	0.33	0.56
Smoke, n (%)	51 (75.0)	39 (69.6)	0.44	0.51
Hypertension, n (%)	49 (72.1)	35 (62.5)	1.28	0.26
Diabetes, n (%)	14 (20.1)	14 (25.0)	0.34	0.56
Rheumatic heart disease, n (%)	13 (19.1)	10 (17.9)	0.03	0.86
Rutherford stages				
IIa	33 (48.5)	23 (41.1)	0.69	0.40
IIb	35 (51.4)	33 (58.9)		
Death within 30 days, n (%)	3 (4.4)	2 (3.5)	–	1

### Surgical methods

All patients were diagnosed with ALI according to their history of sudden onset, disappearance of arterial pulse, presence of low temperature limb, as well as color doppler ultrasonography and CTA. Accompanied by arteriosclerosis was diagnosed according to Medical history of a cold-blooded limb, intermittent claudication and computed tomography (CT) scan. Once diagnosed, if there is no anticoagulant contraindication, we will use low molecular heparin for anticoagulant therapy to prevent secondary thrombosis, and prepare for emergency surgery. Group A: 70 limbs underwent ipsilateral arterial thrombectomy via the Fogarty catheter to take out the mixed and secondary thrombus. After the proximal artery received a good blood jetting and the distal artery received a good blood return, the artery was sutured. The postoperative anticoagulation and disaggregation were performed. Electrocardiogram (ECG) monitoring was performed, the blood circulation condition of the injured limb was observed carefully.

Group B: 59 limbs underwent ipsilateral arterial thrombectomy and DSA at the same time. The suitable double-lumen Fogarty catheter was inserted into the target artery under the guidance of a guidewire. If the DSA showed poor blood flow in the outflow tract after the thrombectomy, the catheter was retained for 24–48 h in the artery with residual thrombus fragments. The following conditions revealed by postoperative DSA should perform the second intervention: (1) the flow-limiting dissection of the artery formed after thrombectomy; (2) the potential arteriosclerosis stenosis rate > 50%; (3) stenting was performed for non-thrombotic arterial dissection; (4) intracavitary repair with coated stent was performed for limb aneurysms.

### Evaluation of therapeutic efficacy

Efficacy evaluation: Clinical success is defined as: The resting pain of the affected limb disappeared after surgery, The Rutherford scale improved by at least 1 grade from pre-operation [9]. The success rate of the primary surgery, the second intervention rate, the amputation rate, and the mortality rate within 30 days postoperation were assessed.

### Statistical analysis

All data were analyzed using SPSS 24.0 software and expressed as a component ratio in this study. The differences in these parameters between the two groups were compared using the chi-square test. When comparing the differences in these parameters between the two groups, the *t* test is used for the continuous numeric data of normal distribution, and the Chi-square test is used for the categorical data. *P* < 0.05 indicated statistically significant.

## Results

In group A, there were 58 cases of arterial embolism and 12 cases of arteriosclerosis with thrombosis. In group B, there were 51 cases of arterial embolism and seven cases of arteriosclerosis with thrombosis.

### The comparison of one-stage blood flow recovery between the two groups

The blood flows of 54 limbs (54/70, 77.1%) in Group A and 56 limbs (56/58, 96.6%) in Group B were improved after the primary surgery. In group B, 41 of 58 limbs did not receive a second intervention, 13 of 58 limbs received the stent implantation in the same period, and 2 of 58 limbs were indwelled thrombolytic catheters. There was a significant difference in the one-stage blood flow recovery between the two groups (*P* < 0.05).

### The comparison of the second intervention rate between the two groups

In group A, the blood flows of 16 limbs were not improved or even re-aggravated after improvement. Among them, 14 of 16 limbs underwent a second intervention on the second day after the surgery and 2 of 16 limbs remained untreated. The DSA showed that 2 of 16 limbs had residual thrombosis of distal arterioles and a poor outflow tract. After simple catheter thrombolysis, their blood flows were improved and the popliteal pulses were returned to normal after 2–3 days. The DSA also showed that 6 of 16 limbs had an arterial flow-limiting dissection and another 6 of 16 limbs had severe hardening and narrowing of the arteries. After stent implantation, the blood flows of these 12 limbs were recovered. In Group B, the blood flows of 3 limbs were not improved after the primary surgery. These three patients received a second intervention and 2 of them were improved. The second intervention rate of Group B (3/58, 5.2%) was lower than that of Group A (14/70, 20%) (*P* < 0.05).

### The comparison of the amputation rate between the two groups

There were four and two patients underwent amputation in Group A and Group B, respectively. Among the 2 amputated patients in Group B, they underwent amputation due to the no improvement after the second intervention. The amputation rate of Group A (4/70, 5.7%) was higher than that of Group B (1/58, 1.7%) (*P* < 0.05).

### The comparison of the mortality rate between the two groups

In Group A, three patients died, and in Group B, two patients died within 30 d after the surgery. In Group A, two patients died from multi-organ failure and one patient died from an aortic dissection. In Group B, 1patient died from heart failure and one patient died from a cerebral hemorrhage. There was no significant effect of the mortality rate between Group A (3/68, 4.4%) and Group B (2/56, 3.6%) Table 2.

**Table 2:** The ischemic plane and therapeutic efficacy of the limb (128limbs)

n (%)	Group A (n = 70)	Group B (n = 58)	*t*/${x}^{2}$	*P*
Common femoral artery and above	9 (12.9)	8 (13.8)	0.69	0.40
Superficial femoral artery	21 (30.0)	14 (24.1)	0.55	0.46
Popliteal artery	26 (37.1)	21 (36.2)	0.01	0.91
Inferior genicular artery	14 (20.0)	15 (25.9)	0.62	0.43
Success of the primary surgery	54 (77.1)	56 (96.6)	9.89	0.002
Second intervention	14 (20.0)	3 (5.2)	6.05	0.01
Amputation	4 (5.7)	1 (1.7)	1.46	0.22

## Discussions

ALI occurs suddenly and rapidly, and if not treated timely and effectively, limb necrosis may occur within a few days, even threatening patients’ lives. It is not difficult to make a diagnosis based on an acute medical history and clinical symptoms. The preoperative CTA examination is more helpful to clarify the characteristics of the lesion, such as the length and range of the thrombus, with or without atherosclerosis, aneurysm, or dissection. In this study, the etiologies of the patients were mostly as follows: (1) acute lower limb arterial embolization, which was caused by the cardiogenic thrombus due to arrhythmia or atrial fibrillation, aortic mural thrombus, or aneurysm thrombus; (2) acute lower limb arterial thrombosis, which was caused by the segmental arterial stenosis and occlusion, arteritis, or hypercoagulable status due to polycythemia vera and thrombocytosis; (3) arterial dissection, which was caused by the increased compression of the arterial false lumen to the arterial true lumen, or the cover of a limb artery by the intima translocation. Thus, the treatments for ALI should be different according to its different etiologies.

**Fig. 1:**
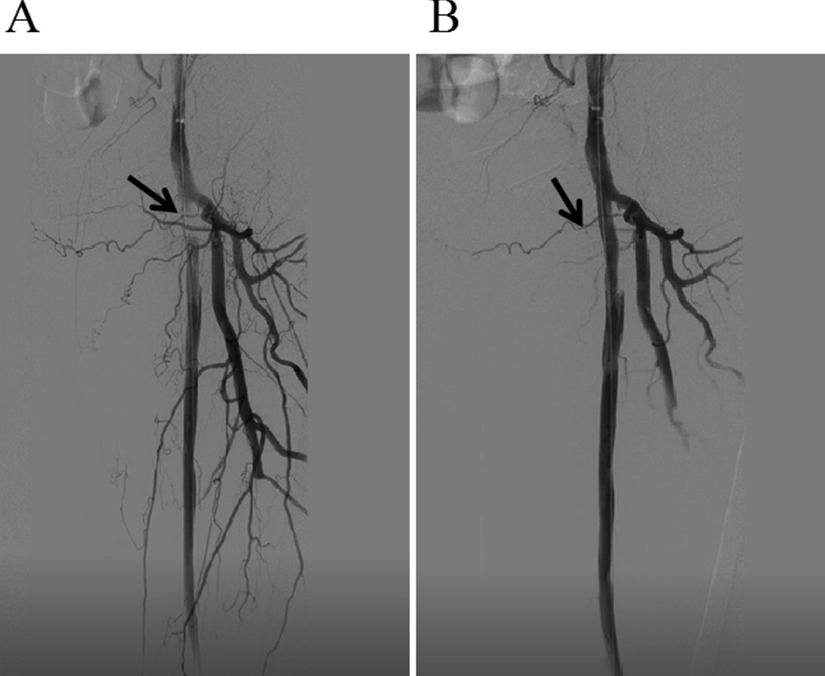
A patient with current limiting dissection formation of superficial femoral artery after embolectomy. **A** The angiography showed the current limiting dissection formation. **B** The DSA showed that the blood flow was recovered after the stent implantation

**Fig. 2:**
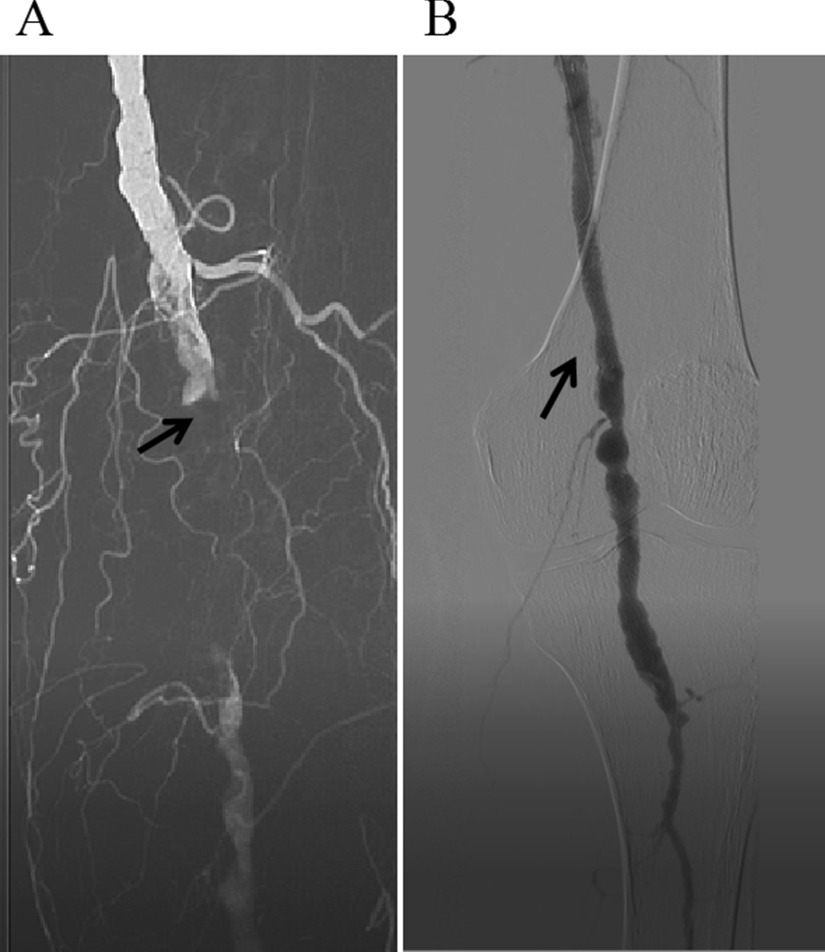
A patient with lower limb arterial embolization accompanied by arteriosclerosis occlusion. **A** The DSA showed the incompletely removed and residual embolus. **B** The DSA showed that the blood flow was recovered after thrombolysis and stent implantation

**Fig. 3:**
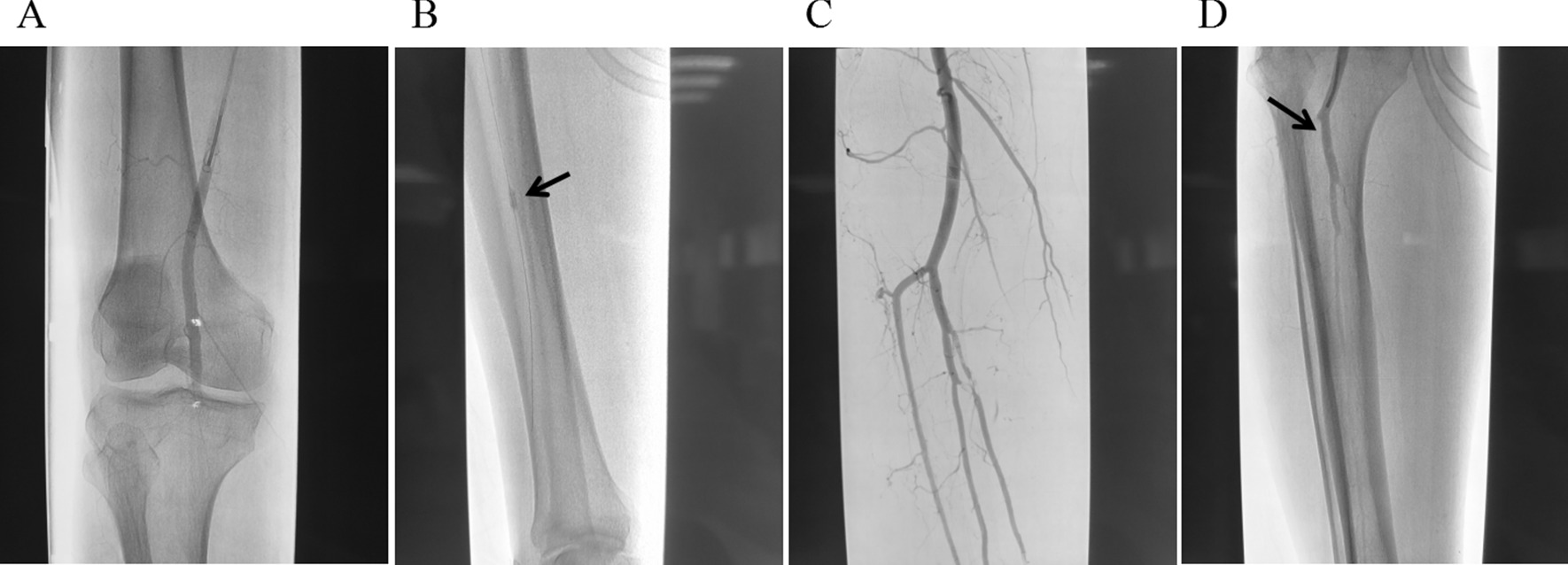
A patient with popliteal artery embolization below P3, anterior tibial and posterior tibial artery thrombosis. **A** The DSA showed the popliteal artery embolization below P3 with anterior tibial and posterior tibial artery thrombosis. **B** The DSA showed that the V18 guide-wire guided the Fogarty tube to remove the posterior tibial artery thrombus. **C** The DSA showed the defect of the anterior tibial artery. **D** The DSA showed that the blood flow of the inferior genu artery was recovered after thrombectomy of the anterior tibial artery

In 1963, Fogarty invented the thrombectomy catheter [10], which created a new situation of surgical thrombectomy for ALI, especially for arterial embolism. The arterial thrombectomy via the thrombectomy catheter has been continued to this day, while its disadvantage is the high incidence of I/R injury. The CDT is also an effective treatment for ALI [11, 12]. Due to the relatively slow recovery of blood supply, the incidence of I/R injury after CDT is low. CDT is not the first choice for patients at stage IIb with arterial embolization, in particular for patients with long onset time, since their embolus are mostly white thrombus which is difficult to be dissolved. Besides, CDT is not the first choice for patients with polycythemia vera, because it can prolong the ischemia of the affected limb and cause irreversible injury. Percutaneous mechanical thrombectomy devices have been proposed over the past 2 decades to speed up the time required for dissolution of thrombus, more and more medical institutions are trying to use these devices. But many medical institutions like our hospital don’t have these advanced devices yet [13]. The thrombectomy is recommended for the preferred choice for ALI patients without aortic dissection. In the current study, the blood supply of the affected limb of 77.1% of patients in Group A was recovered after the primary surgery, suggesting the effectiveness of arterial thrombectomy. Nevertheless, the limitation of simple thrombectomy is that the success criterion is considered as a good blood returning from the distal artery and a good blood jetting from the proximal artery. However, it is impossible to accurately determine whether the blood flow between the proximal and distal artery is improved, or whether the artery is narrowed or injured [14]. In this study, there were 3 main reasons for 20% of patients in Group A whose blood supply did not recover after the primary surgery and underwent a second intervention. The first reason was that thrombectomy resulted in the injury of the arterial intima and the formation of flow-limiting dissection (Fig. 1), which was mostly seen in patients with local segment artery stenosis, excessive balloon pressure of thrombectomy, or repeated thrombectomy. The second reason was that the main artery thrombus was not totally removed, and there were branch arteries supplying blood above the embolized segment. The most cases were that the thrombectomy tubes entered the branch or penetrated the artery wall, failing to reach the far segment of the main artery. Besides, there were also some cases with incomplete thrombectomy due to arteriosclerosis and stenosis. The third reason was that the blocked distal outflow tract caused by unsuccessful thrombectomy, most of which were due to the thrombectomy tube failing to pass the superficial femoral and popliteal arteries to the inferior genicular artery. For those who have undergone the failed arterial thrombectomy, the second intervention under DSA was recommended instead of the second thrombectomy, since most of the detachable thrombus can be removed in the primary thrombectomy and the secondary thrombus formation was prevented by the postoperative anticoagulation. The second thrombectomy could not solve this problem, but increased the injury caused by thrombectomy. In the present study, among these patients underwent second intervention in Group A, six cases were the iatrogenic injury with limiting-flow dissection formation, and the thrombus was removed after the stent implantation; 3 cases had the latent arterial stenosis with residual thrombectomy and were relieved by balloon dilatation and stent placement 1 day after thrombolysis with catheterization (Fig. 2).The selective thrombectomy was performed under V18 guidewire fluoroscopy for those patients who were diagnosed with the blocked outflow tract that caused by thrombosis in the lower three branches of the knee using DSA (Fig. 3).

**Fig. 4:**
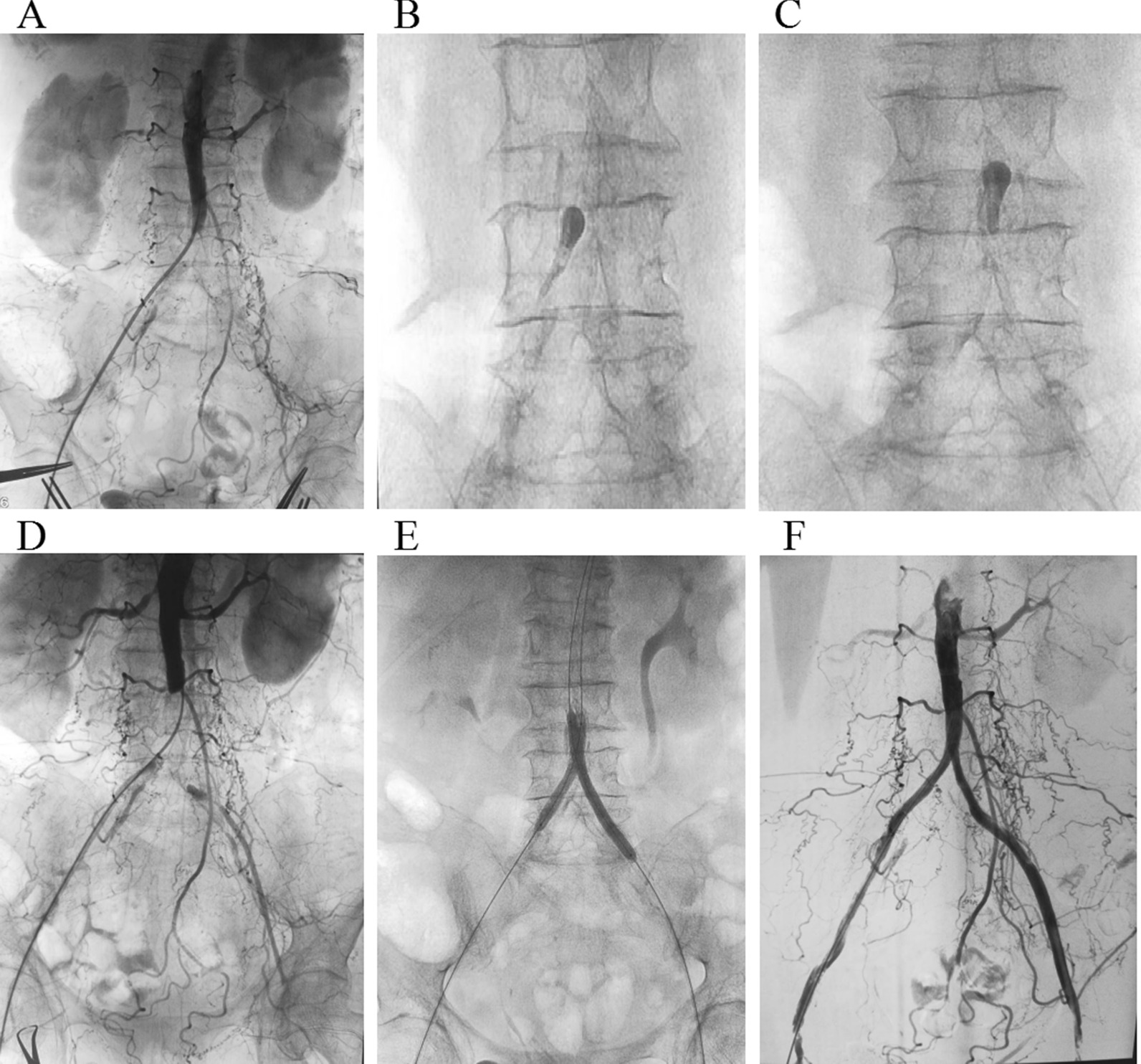
A patient with lower limb arterial embolization with primary iliac artery stenosis. **A** The DSA showed the lower limb arterial embolization with primary iliac artery stenosis. **B** The thrombectomy balloon deforms after passing through the narrow right iliac artery under DSA. **C** The thrombectomy balloon deforms after passing through the narrow left iliac artery. **D** Poor blood flow in the main iliac artery after thrombectomy. **E** Bilateral iliac artery simultaneous balloon dilation. **F** Blood flow was recovered after bilateral iliac artery stent placement

The intraoperative concomitant angiography can determine the characteristics of the lesion, the length of the thrombus segment, with or without arteriosclerosis, stenosis, occlusion, aneurysm, or arterial dissection. The twisted and narrow arterial segment can be selectively passed under the guidance of the guidewire, to avoid injury of the aorta wall and branch arteries caused by embolization catheter. For the thrombosis of the three branches of the lower knee, it can also be selectively cleared, greatly improving the outflow tract. The routine arteriography after thrombectomy is helpful to improve the success rate of operation and to discover the thrombus clearance, the filling of the proximal and the distal arterial lumen, the velocity of blood flow, and the presence or absence of latent arterial stenosis or the secondary iatrogenic injury of the arterial intima [15, 16]. Successful thrombus removal followed by near or complete revascularization was considered the sign of success. Currently, many advance and exquisite devices could achieve near-complete revascularization by eliminating thromboembolus and mending the underlying lesion at the same time, and they have been shown to be safe and effective [17, 18]. We share the same goal in performing a successful surgery even devoid of such advanced devices. Once diagnosed (Fig. 4), these conditions can be managed timely, significantly reducing the failure rate of operations. Compared with the single cavity tube, the disadvantages of double-cavity thrombectomy tube for thrombectomy under intervention include the larger resistance for balloon filling, the slower regulation of balloon size and pressure, and the poor operating flexibility. During the operation, the filling pressure should be controlled according to the pulling resistance and the balloon size under fluoroscopy. The thrombus cannot be removed if the balloon is too small, while the intima will be injured if the balloon is too large. Especially, when the balloon entered the popliteal artery from the tibial artery, the catheter should be retracted slowly after the balloon filling was adjusted with a short pause under fluoroscopy to avoid residual thrombosis. The presence or absence of stenosis can be determined according to the morphology and resistance of balloons when the catheters were retracted under fluoroscopy. The size of the balloon should be adjusted timely in case of stenosis to avoid the formation of dissection caused by the intimal injury. If the angiography revealed that the arterial stenosis was > 50%, balloon dilatation and stent placement should be performed simultaneously. Thrombus shedding in limb aneurysms is a common cause of recurrent ALI. The presence of proximal aneurysm should be considered in patients with non-arrhythmia and lower limb arterial embolization. In this study, there was one patient with popliteal aneurysms who had a history of lower limb ischemia and thrombolysis for several times. The covered stent Viabahn and thrombectomy were performed on this patient in the same period to isolate tumors and no recurrence was occurred in the follow-up for up to 3 years. For the patients with lower limb ischemia caused by aortic dissection (AD), the key to the success of the operation is the intra-lumen repair of arterial dissection and the recovery of true lumen blood supply. Most of the patients with thrombectomy failure and postoperative death were owing to that the AD was not confirmed before the operation. Patients with ALI were combined with basic diseases in this study. There was no significant difference in the death rate between Group A and B within 30 days after the operation.

## Conclusions

Since ALI is a dangerous disease, the timely and effective diagnosis and treatment are needed. Arterial thrombectomy is an effective surgical method for ALI, and intracavitary interventional therapy is an effective remedy for thrombectomy failures. The DSA combined arterial thrombectomy may elevate the success rate of operation and reduce the amputation rate. However, arterial thrombectomy has limitations such as trauma and residual thrombosis. The long-term follow-up and treatment of the risk factors of secondary ALI, such as organic heart disease-arrhythmia, aneurysm, and atherosclerosis, should be conducted to improve the long-term blood flow and survival rates of patients.

## Data Availability

The raw data may be made available upon reasonable request from the corresponding authors.

## Abbreviations

DSA: Digital subtraction angiography
ALI: Acute lower limb ischemia
CDT: Catheter contact thrombolysis
PMT: Percutaneous mechanical thrombolysis
I/R: Ischemia/reperfusion injury
CTA: Computed tomography angiography
CLI: Critical limb ischemia
CT: Computed tomography
ECG: Electrocardiogram
AD: Aortic dissection
